# Starting with 24-h levodopa carbidopa intestinal gel at initiation in a large cohort of advanced Parkinson’s disease patients

**DOI:** 10.1038/s41598-024-54299-z

**Published:** 2024-02-14

**Authors:** Szabolcs Szatmári, József Attila Szász, Károly Orbán-Kis, Simona Bataga, Marius Ciorba, Előd Nagy, Radu Neagoe, István Mihály, Péter Zsombor Szász, Krisztina Kelemen, Attila Frigy, Andrea Csipor-Fodor, Viorelia Adelina Constantin

**Affiliations:** 1 2nd Clinic of Neurology, Târgu Mures County Emergency Clinical Hospital, Târgu Mureș, Romania; 2 University of Medicine, Pharmacy, Science and Technology of Târgu Mures, George Emil Palade, Gh. Marinescu Street No 38, 540142 Târgu Mures, Romania; 3 Department of Gastroenterology, Târgu Mures County Emergency Clinical Hospital, Târgu Mures, Romania; 4 Laboratory of Medical Analysis, Clinical County Hospital Mures, Târgu Mures, Romania; 5 2nd Clinic of Surgery, Târgu Mures County Emergency Clinical Hospital, Târgu Mures, Romania; 6 Department of Neurology, Emergency County Hospital, Miercurea-Ciuc, Romania; 7 Department of Internal Medicine IV, Clinical County Hospital Mures, Târgu Mures, Romania

**Keywords:** Neurology, Neurological disorders, Parkinson's disease

## Abstract

Continuous intra-jejunal infusion of levodopa-carbidopa intestinal gel (LCIG) is a long-term proven and effective treatment in advanced Parkinson’s Disease (APD). Efficacy and safety of 16-h administration of LCIG has already been established. Additional benefits of 24-h LCIG administration have been reported in several case series and small clinical studies. The aim of this retrospective study was to compare the characteristics of patients who needed 24-h LCIG from the beginning of the DAT (device-aided treatment) with those who remained with the standard 16-h LCIG treatment and to identify particular motives if any. We initiated LCIG in 150 patients out of which in case of 62 patients (41,3%) due to unsatisfactory initial clinical benefits continuous 24-h LCIG was deemed necessary. Despite the subjective complaints and more severe clinical condition, at baseline evaluation we found statistically significant differences between 16-h LCIG cohort and 24-h LCIG cohort only in case of incidence of freezing (47% vs 65%, p = 0.03) and sudden off (32% vs 48%, p = 0.04). Wake hours/daytime LCIG does not always sufficiently improve the patient's quality of life in some patients due to persistent nighttime troublesome symptoms. Instead of labeling the patient as a non-responder, it is worth trying the 24-h LCIG dosage in a carefully selected group of patients, as there is currently no consensus on reliable criteria that serve the decision in these patients.

## Introduction

Parkinson’s disease (PD) is the second most common neurodegenerative disorder after Alzheimer’s disease and is the most common type of parkinsonism^1^. Among the drugs approved for the symptomatic treatment of PD, the most effective method for restoring dopaminergic tonus is substitution therapy with levodopa (LD) formulations, which remains the gold standard^2,3^. Although LD is long-term efficacious and well tolerated, its clinical utility over time is often limited by the development of troublesome motor complications: response fluctuations (wearing-off and other motor fluctuations), and LD-induced dyskinesias, dystonias^4^. In patients with advanced Parkinson's disease (APD) these complications cannot be managed using traditional oral and transdermal treatment options (combination of LD and different add-on options); therefore, the quality of life (QoL) of these patients is continuously deteriorating^5,6^. Continuous intra-jejunal infusion of levodopa-carbidopa intestinal gel (LCIG) is a long-term proven and effective treatment in APD with severe motor fluctuations, with or without dyskinesias. Efficacy and safety of 16-h of continuous administration of LCIG in reducing motor fluctuations and ameliorating different nonmotor symptoms has been established in multiple prospective studies and reviewed extensively^7–10^. The usual treatment duration is typically 16 wake-hours, the dosages are calculated according to formulas based on experience with this timeframe^11,12^. Day-time and night-time LCIG administration are also possible when there is a medically justified reason. Patients’ characteristics and further/additional benefits of 24-h LCIG administration have been reported in several case series and small clinical studies.

Based on the current experience the 24-h LCIG should be recommended for those patients who present one or more of the following complaints and signs, without any or only partial benefit after 16-h LCIG: severe night-time akinesia/bradykinesia, severe early morning akinesia and /or delayed morning latency (delayed time to functional “on” state following the first morning LD dose), unresponsive freezing of gait and falls prevention, painful dystonia, stiffness due to wearing off symptoms overnight, severe night-time urinary problems, sleep fragmentation/poor sleep quality, significant caregiver burden at night-time, poorly controlled troublesome dyskinesias, diphasic dyskinesias^13–15^.

The benefits and safety of long-term administration of LCIG therapy in patients with severe motor fluctuations and troublesome, severe dyskinesias were presented in a previous work^16^.

## Study question, study design, measures and outcomes

The aim of this retrospective study was to compare the characteristics of patients who needed 24-h LCIG from the beginning of the DAT with those who remained with the standard 16-h LCIG treatment and to identify particular motives if any.

We evaluated all APD patients that started LCIG therapy in our tertiary center, between June 2011 and June 2021.

In previous publications, we detailed our procedure for selection of eligible patients for LCIG therapy, the way to evaluate different motor complications, the challenges of the testing process and establishing effectiveness (exclusion of patients considered non-responders), all under the specific conditions of clinical practice in our country^17–19^.

In all cases therapy was initiated based on the evaluation by a large multidisciplinary team that included neurologists from the Neurology Clinic in Târgu Mureș, Romania. In this center, during the examined period LCIG was the only available device-aided therapy. The time between the initial presentation of this therapeutic option and the start of LCIG testing was recorded. Testing the efficacy of LCIG therapy and also the titration of the optimal dosage was done during continuous hospitalization. Calculation of estimated LCIG doses was performed according to literature recommendations^11^. In order to maximize therapeutical benefit, we continuously adjusted LCIG doses, not only during the titration period but also after performing the PEG-J. Besides recording the demographic parameters of our patients and the clinical aspects such as disease duration, treatment duration and the spectrum of different motor complications (based on 24-h patient diaries) we appreciated the severity (both during on and off phase) as measured on the Hoehn and Yahr scale, and on the MMSE (Mini-Mental State Evaluation) score. We also recorded the doses of all medications (LD, dopamine agonists, monoamine oxidase B inhibitor (MAO-Bi), catechol-O-methyl transferase inhibitor (COMTi) and/or amantadine) as well as motor fluctuations and dyskinesias. At discharge patients were asked to assess their subjective improvement of PD symptoms using the Patient Global Impression of Improvement (PGI-I) scale; Our results regarding this scale were already described in our previous publication^20^.

Statistical analysis was performed using the Prism 8.0 software package (GraphPad Software, San Diego, CA, USA). Normality of data pools was tested each time. Depending on the type of data descriptive statistics, parametric or non-parametric t-tests and Kruskal–Wallis test were used. For contingency tables Fisher’s exact test was performed. The level of statistical significance was p < 0.05. Each patient signed the institution's informed consent (data use agreement, agreement for filming). Furthermore, the study was approved by the local ethical committee (UMFST 94/19.05.2017).

## Results

**Figure 1:**
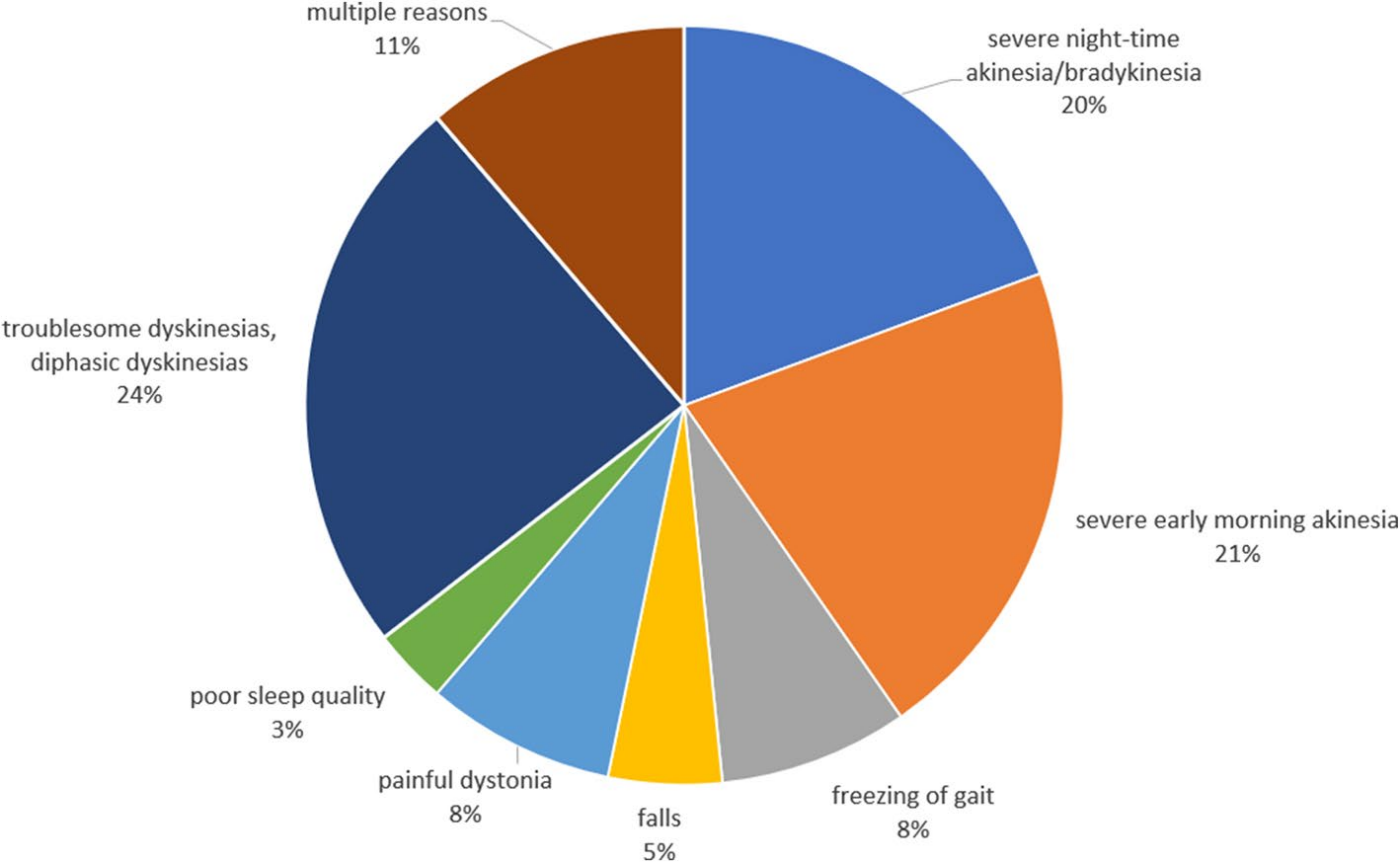
Main reasons for 24-h administration of LCIG.

During the aforementioned 10 years period we initiated LCIG treatment in a total of 150 patients out of which in the case of 62 patients (41.3%) after careful post-PEG-J consideration, 24-h LCIG was deemed necessary. The clinical reason for continuous around-the-clock LCIG treatment was unsatisfactory initial control of some motor complications (Table 1). The baseline characteristics of patients are presented in Table 2. Patients treated with 24-h LCIG presented a significantly more severe clinical picture both in the on and off phase, respectively lower MMSE scores; they were also treated with significantly higher doses of levodopa. The spectrum of motor complications (fluctuations and different subtypes of dyskinesia) is presented in Table 3. Despite the subjective complaints and the apparent more severe clinical condition, we only found statistically significant differences (more severe in the group of patients treated with 24-h LCIG) in case of freezing of gait and sudden off. The main reasons why we opted for the 24-h administration of LCIG are presented in Fig. 1.

**Table 1:** Characteristics suggesting the need for 24-h LCIG in patients with APD.

Severe night-time akinesia/bradykinesia
Severe early morning akinesia and /or delayed morning latency (delayed time to functional “on” state following the first morning LD dose)
Unresponsive freezing of gait and falls prevention
Painful dystonia
Stiffness due to wearing off symptoms overnight
Severe night-time urinary problems
Sleep fragmentation/poor sleep quality
Significant caregiver burden at night-time
Poorly controlled troublesome dyskinesias, diphasic dyskinesias

**Table 2:** Baseline characteristics of APD patients treated with LCIG.

Characteristics	Daytime (16–18-h LCIG) N = 88	24-h LCIG N = 62	p
Men, n (%)	46 (52%)	35 (56%)	ns
Women, n (%)	42 (48%)	27 (44%)	ns
Age, years (mean ± SD)			
All	63.2 ± 8.6	65.1 ± 7.4	ns
Men	61.9 ± 9.2	65.1 ± 7.9	ns
Women	64.6 ± 7.7	65.1 ± 6.8	ns
Time since PD diagnosis (years)			
Mean ± SD	10.7 ± 4.3	11.3 ± 4.7	ns
Median	11	10	
Hoehn-Yahr scale, before PEG			
“On” state			
Median	3	3	< 0.03
Mean	3.1 ± 0.3	3.4 ± 0.5	
“Off” state			
Median	4	5	< 0.03
Mean	4.3 ± 0.5	4.5 ± 0.5	
MMSE score (mean ± SD)	26.4 ± 2.4	25.7 ± 2.4	0.041
Levodopa:			
Treatment duration (years)	10.3 ± 4.2	11.1 ± 4.6	ns
Average dose (mg/day)	814.9 ± 257.5	909.9 ± 250.7	0.03
Median dose (mg/day)	750	918.8	ns
Dose frequency, x/day	5.1 ± 0.9	5.3 ± 1.1	
Dose frequency, x/day, median	5	5	
Dopamine agonists (n,%)	73 (83%)	49 (79%)	ns
Pramipexole (n; average mg)	24; 2.2 ± 0.6	18; 2.3 ± 0.6	ns
Ropinirole (n; average mg)	26;13.2 ± 4.8	7; 14.3 ± 6.0	0.009
Rotigotine (n; average mg)	26; 7.9 ± 2.9	25; 8.4 ± 3.0	ns
MAO-Bi (n,%)	59 (67%)	42 (68%)	ns
COMTi (n,%)	52 (59%)	35 (56%)	ns
Amantadine (n,%)	23 (26%)	18 (29%)	ns
LCIG acceptance in less than 30 days (n;%)	42 (48%)	33 (53%)	ns
LCIG calc (mean ± SD)	1271 ± 309.1	1361 ± 333.6	ns

*APD* advanced Parkinson's disease, *LCIG* levodopa-carbidopa intestinal gel, *SD* standard deviation, *n and N* number of patients, *MMSE* Mini-Mental State Examination, *MAO-Bi* monoamine oxidase B inhibitor, *COMTi* catechol-O-methyl transferase inhibitor, *LCIG* acceptance the time (in days) between the presentation of the therapeutic option and the start of LCIG testing, *LCIG calc* calculated LCIG doses.

**Table 3:** Spectrum of motor complications before LCIG initiation.

Characteristics	Daytime (16–18-h LCIG) N = 88	24-h LCIG N = 62	p
“Off” duration (hours, mean ± SD)	4.5 ± 0.9	4.8 ± 1.2	ns
Peak-dose dyskinesia			
Duration (hours, mean ± SD)	2.9 ± 0.7	3.0 ± 1.0	ns
n%	56 (64%)	39 (63%)	ns
Diphasic dyskinesia			
Duration (hours, mean ± SD)	3 ± 0.9	3.0 ± 0.8	ns
n%	26 (30%)	18 (29%)	ns
Dystonia (hours, mean ± SD)	1.8 ± 0.7	1.8 ± 0.8	ns
n%	21 (24%)	10 (16%)	ns
Early morning akinesia			
Years, mean ± SD	2.3 ± 1.5	2.8 ± 1.9	ns
n,%	74 (84%)	58 (94%)	ns
Delayed “on”			
Years, mean ± SD	2.2 ± 1.3	2.8 ± 1.6	ns
n,%	49 (56%)	41 (66%)	ns
“No on”			
Years, mean ± SD	1.9 ± 1.0	1.9 ± 0.9	ns
n,%	16 (18%)	19 (31%)	ns
“Sudden off” (n,%)	28 (32%)	30 (48%)	0.04
“Freezing”			
Years, mean ± SD	2.0 ± 0.9	1.9 ± 0.9	ns
n,%	41 (47%)	40 (65%)	0.03

*APD* advanced Parkinson's disease, *LCIG* levodopa-carbidopa intestinal gel, *SD* standard deviation, *n and N* number of patients.

## Discussion

Parkinson's disease (PD) is a progressive neurodegenerative disorder with various motor and non-motor symptoms. Mitochondrial dysfunction, aging and synuclein aggregation, neuroinflammation and increased oxidative stress all play a role in its complex etiopathogenesis^21,22^. Substitution therapy with LD is still key for the best clinical improvements at all stages of the disease. Long-term LD administration, the continuous progression of nigrostriatal degeneration and pulsatile drug delivery are considered risk factors for the development of motor and non-motor complications. These complications contribute to impairments in health-related quality of life, caregiver burden and psychosocial functioning. Motor fluctuations and dyskinesias can be reduced, although the efficacy of different drug combinations varies. Many of the new add-on options, such as the new peripheral catechol-O-methyltransferase inhibitor opicapone^23^ and the third generation, reversible monoamine oxidase B inhibitor and glutamate modulator safinamide^24,25^ are not yet available in Central and Eastern Europe, or indeed in our country^26^.

When the motor complications can no longer be improved to an acceptable level, it is necessary to evaluate the patients in order to introduce device-aided therapies (enteral infusions of levodopa, subcutaneous infusion of apomorphine or deep brain stimulation)^27,28^. The levodopa/carbidopa intestinal gel is a treatment developed for patients with APD. The LCIG delivered continuously into the upper intestine via a percutaneous endoscopic gastro-jejunostomy (PEG-J), by bypassing the very common gastroparesis in APD^29,30^, provides more stable plasma levels (continuous dopaminergic stimulation) compared to oral LD^31^. Timely introduction of LCIG therapy may improve the QoL of APD patients that are on optimized oral/transdermal therapy but have complications or a poorly controlled clinical picture. The daytime 16-h LCIG infusion is efficient, safe and improves QoL, motor and non-motor symptoms in APD patients. In parallel, it allows reducing pill-burden, thus increasing treatment compliance^32,33^. Additional benefits have been reported when used as a continuous 24-h LCIG infusion^34–36^. However, a comprehensive expert opinion report on the practical aspects of 24-h LCIG administration was only published in 2021^37^. The recently published sub-analysis of the COSMOS study (COmedication Study assessing Mono- and cOmbination therapy with levodopa-carbidopa inteStinal gel) demonstrated that 24-h LCIG led to improvements in specific motor symptoms, as well as non-motor symptoms and treatment-related symptoms in patients with APD who were treated with LCIG for ≥ 12 months, with safety findings consistent within the known LCIG profile^38^.

Beyond the usual recommendations mentioned in Table 1, further significant improvements were observed for non-motor symptoms, specifically in sleep/fatigue, mood/ cognition, hallucination, urinary problems. These overall improvements in sleep quality, daytime sleepiness, and fatigue are consistent with improvements in night-time akinesia/bradykinesia^35^.

It should be emphasized that most of the cases reported in the literature refer to the transition from daily administration (16 h) to continuous (24 h). The previously mentioned review provides practical advice on the management of patients transitioning to 24-h LCIG infusion. The proportion of these patients can reach up to 40% according to data from the literature^37^.

In this current work we evaluated the characteristics of a special category of patients with APD, in which, in order to obtain an adequate control of some motor and non-motor complications, we had to use, from the beginning, continuous 24-h administration of LCIG.

We are not aware of any publications that have analyzed a comparably large number of real-life cases (usually more severe cases compared to patients enrolled in targeted clinical trials). The novelty of our study is also reinforced by the lack of clear recommendations of experts regarding the 24-h administration (the use of the different add-on options followed by the progressive increase of the administration duration from 16 to 18 h, as well as later extension to 24 h). As such, the data that emerge from such analyses can guide the therapeutic attitude of the treating physicians.

From the 150 consecutive cases evaluated over the 10-year period, in 62 patients (41.3%) we considered that in order to obtain a significant improvement of motor and non-motor complications, it was necessary to use 24-h LCIG administration in the testing period.

In our previous publications, we analyzed in detail the national regulations and the specific aspects of the activity of the multidisciplinary team for initiation of LCIG therapy^20,39–41^. In this article we analyze the data obtained through the prism of these conditions.

As a principle, we consider that during the titration period on the naso-jejunal tube (carried out under conditions of continuous hospitalization and strict observation) the symptoms are expected to be improved under conditions of daytime administration (16-h LCIG) and only after trying different add-on options (including off-label use of certain medications) should one consider switching to 24-h LCIG^37,42,43^. As such, recommendations include usage of extra doses when stopping the pump and flushing in the evening or increasing the infusion time from 16- to 18-h. As add-on therapeutical option LD preparations with extended release are mentioned or even levodopa/carbidopa/entacapone may be tried before administering a dopamine agonist. Transdermal patches of rotigotine or extended-release formulations of other dopamine agonists (if they have not been used previously, they are well tolerated and there are no contraindications) can also be effective options in relieving nocturnal complaints. From our previous publications, however, it emerges as a main characteristic of our patients with APD that regarding the last dopaminergic treatment prior to the evaluation for eligibility for LCIG, the proportion of dopaminergic agonists (DA) was significantly higher (82%) compared to the literature data (in some cases the combination of two DAs was even used, with the rotigotine patch as an add-on medication)^20,40^. Thus, the "maneuvering space" of the clinician is severely restricted. Also, LD preparations with prolonged release are not constantly available.

An other important practical aspect is the fact that the titration on the naso-jejunal tube is done under conditions of continuous hospitalization (with all the inherent advantages and disadvantages), which for easy to understand reasons is limited in time. In other words, both the decision to complete the test phase (performing PEG-J) and the subsequent adjustments, respectively the establishment of the administration mode (16-h or 24-h) must be done in a relatively short timeframe. Important in this context is the subjective aspect of the patient's perception of the effectiveness of LCIG therapy (unrealistically high expectations can create a false impression of inefficiency and the patient could be considered a non-responder). The statistically significant (albeit subjective) PGI-I score further underlines that “forcing” the 24-h LCIG administration was justified (Table 4).

**Table 4:** Post-PEG evaluation of patients treated with LCIG.

Characteristics	Daytime (16–18-h LCIG) N = 88	24-h LCIG N = 62	p
Hoehn–Yahr scale, after PEG			
“On” state			
Median	3	3	ns
Mean	2.9 ± 0.2	3.0 ± 0.1	
“Off” state			
Median	4	4	ns
Mean	3.8 ± 0.4	3.8 ± 0.4	
PGI-I (mean ± SD)	1.5 ± 0.5	1.9 ± 0.5	0.0047
Median	2	2	ns

*PGI-I* Patient Global Impression of Improvement scale.

Analyzing the main reasons considered decisive for the administration of LCIG on a 24-h basis, it turns out that severe night-time akinesia/bradykinesia and early morning akinesia as well as sleep disorders seem the most influential^37^. Figure no. 1. presents the main reasons which, following repeated evaluations and adjustments, forced us to opt for the 24-h administration of LCIG in order to obtain an improvement in the clinical picture. We specify that all patients with nocturnal akinesia or severe morning sickness were previously treated with DA, a treatment that was maintained during the titration period. In this context, it should be stated that despite all the efforts made, the sleeping conditions during hospitalization can be very different from those at the patient's home. Our attempt to create a profile of the patient with APD who could benefit additionally from the administration of LCIG for 24 h is hampered by the fact that early morning akinesia occurs in the majority of patients in the evaluated group (84% in those with 16 h LCIG and 94% in those with 24 h LCIG, with the difference not being statistically significant). The fact that there is no big difference between the two groups suggests that it is not always easy to predict from the pre-LCIG state whom morning akinesia will respond to 16 h LCIG and whom dyskinesia will need 24 h LCIG to be controlled. Thus, it requires a more individual assessment and not necessarily one size fits all to reach to a decision. There could be also very specific situations such as a troublesome dyskinesia related to flushing the tube before stopping the LCIG pump. This phenomenon was observed by us even during the testing phase of the naso-jejunal probe. Also, it was observed in 2 patients with biphasic dyskinesia, hence the decision to test 24-h LCIG administration^19^.

At the same time, we emphasize that both our own data and the data from the literature suggest that, as a whole, treatment with LCIG is initiated in patients at a more advanced stage of the disease compared to the experts' recommendations^6,17,40,44^. However, we encountered the phenomenon of freezing of gait (47% vs 65%) and sudden off (32% vs 48%) significantly more frequently in patients for which we opted for 24-h LCIG administration (Table 2).

In this retrospective evaluation, we analyzed the 10 years’ activity of a University Teaching Hospital with high patients’ turnover, where the only available device-aided therapy for APD is LCIG. In our previous publications we highlighted that PD patients in our department are treated with similar strategies that are used by professionals in other countries^45–47^. Due to the fact that add-on therapeutical options available in Romania are comparable to those in other Central- and Eastern-European countries we believe that this type of analysis will help the clinicians to identify suitable candidate patients for 24-h LCIG treatment regimen. We hope that comparing databases with relevant data from other centers could result in further practical information for the increasingly secure use of 24-h LCIG.

The strengths of this work are represented by the large number of cases from real-life practice, that started treatment during the 10-year period, respectively the rigorous conditions of initiation during continuous hospitalization. The study however has some limitations, such as the increased prevalence of patients with more advanced PD as well as the fact that the presentation of baseline characteristics does not warrant a detailed analysis of outcome. Also, the retrospective data processing did not allow us to analyze some important non-motor symptoms (different sleep disorders, falls, pain) that may significantly affect QoL, as these were not properly recorded in all cases. In this context, an interesting discussion refers to the dilemmas related to the testing phase and establishing suitability for LCIG. Contrary to the opinions expressed by some authors, regarding the possibility of testing the naso-jejunal probe in ambulatory/telemedicine conditions^48^, we consider that the decision/indication to use the 24-h administration requires a thorough clinical observation in conditions of continuous hospitalization (even if this requires a decision made in a relatively short time interval). We do believe there is a need for targeted studies that allow the easy identification of patients with APD symptoms who could not respond to 16-h LCIG treatment, requiring instead 24 h LCIG therapy from the very beginning. These studies should also outline additional long-term follow-up measures and benefits of these patients. In this context, we believe that the new option of continuous intradermal infusion of foslevodopa/foscarbidopa (expected to be administered continuously for 24 h) also fits this trend^49^.

The introduction of LCIG treatment in the current conventional way does not always sufficiently improve the patient's quality of life. Instead of labeling the patient as a non-responder, it is worth trying the 24-h dosing in a carefully selected group of patients, as there are currently no reliable criteria that will help in the decision in every case. In addition to the indications outlined by the experts' recommendations (night-time and early morning akinesia, poor sleep quality, uncontrolled troublesome dyskinesias), we consider that patients who receive higher daily doses of oral LD, present a more severe clinical picture with freezing phenomenon or those who experience sudden off type complications, should have additional benefits following the administration of 24-h LCIG. Nevertheless, the initiation of the 24-h LCIG treatment should be decided individually, after considering the potential benefits and disadvantages.

### Ethical approval

This study enrolled patients admitted to the Neurological Clinics in Târgu Mureş after they were informed about the study. According to national legislation, all patients had to sign the written consent form of the teaching hospital. Furthermore, the study was approved by the Ethics Committee of the University of Medicine and Pharmacy from Târgu Mureș, approval no. 94/19.05.2017 (https://www.umfst.ro/universitate/comisii-de-etica/comisia-de-etica-a-cercetarii-stiintifice/avize/2017.html, accessed on 19 May 2017), which requires all human studies to be conducted entirely in accordance with the Declaration of Helsinki.

### Informed consent

Informed consent was obtained from all subjects involved in the study.

## Data Availability

The data presented in this study are available on request from the corresponding author. The data are not publicly available due to privacy reasons.
